# Association between paraoxonase gene and stroke in the Han Chinese population

**DOI:** 10.1186/1471-2350-14-16

**Published:** 2013-01-28

**Authors:** Guojun Zhang, Wenjin Li, Zhiqiang Li, Hong Lv, Yonghong Ren, Ruimin Ma, Xiaohong Li, Xixiong Kang, Yongyong Shi, Yimin Sun

**Affiliations:** 1 Laboratory Diagnosis Center, Beijing Tiantan Hospital Affiliated to Capital Medical University, Beijing, 100050, China; 2 Bio-X Institutes, Key Laboratory for the Genetics of Developmental and Neuropsychiatric Disorders (Ministry of Education), Shanghai Jiao Tong University, Shanghai, 200030, China; 3 CapitalBio Corporation, 18 Life Science Parkway, Changping District, Beijing, 102206, China; 4 National Engineering Research Center for Beijing Biochip Technology, 18 Life Science Parkway, Changping District, Beijing, 102206, China

**Keywords:** Polymorphisms, Paraoxanase gene, Hemorrhagic stroke, Ischemic stroke, Association

## Abstract

**Background:**

The human paraoxonase (*PON*) gene family has three isoforms: *PON1*, *PON2* and *PON3*. These genes are implicated as potential risk factors of cerebrovascular disease and can prevent oxidative modification of low-density lipoproteins and atherosclerosis. This study evaluated the association between the genetic variants of all three *PON* genes and the risks of total stroke, ischemic stroke and hemorrhagic stroke in the Han Chinese population.

**Methods:**

A total of 1016 subjects were recruited, including 508 healthy controls and 498 patients (328 with ischemic stroke and 170 with hemorrhagic stroke). A total of 11 single nucleotide polymorphisms (SNPs) covering the *PON* genes were genotyped for statistical analysis. Two of the 11 SNPs (rs662 and rs854560) were contextualized in a meta-analysis of ischemic stroke.

**Results:**

The presence of rs705381 (−162) in the promoter region of *PON1* was significantly associated with total stroke (*P*_*adjusted*_ = 0.0007, *OR* = 0.57 [95% CI = 0.41-0.79]) and ischemic stroke (*P*_*adjusted*_ = 0.0017, *OR* = 0.54 [95% CI = 0.37-0.79]) when analyzed using a dominant model, but was not associated with hemorrhagic stroke. There was also a nominal association between rs854571 (−824) and total stroke. Meta-analysis demonstrated a significant nominal association between rs662 and ischemic stroke, but there was no evidence of an association between rs662 and ischemic stroke risk in a single site association study.

**Conclusions:**

These findings indicate that polymorphisms of *PON1* gene may be a risk factor of stroke.

## Background

Stroke is recognized as one of the leading causes of death and severe neurological disability worldwide. Ischemic and hemorrhagic stroke are the two primary subtypes [1]. Data from family-based studies [2], twin studies [3,4], and animal experiments [5,6] indicate that genetic factors play a major role in stroke. A small isolated group of strokes have previously been ascribed to single-gene disorders [7].

Intermediate phenotypes of stroke are seen clinically. Atherosclerosis, as an intermediate phenotype of stroke, has been extensively investigated as a major underlying cause of cardio- and cerebrovascular disease [8-10]. There is also a strong inverse association between high-density lipoprotein (HDL) levels and the development of atherosclerosis, and similar results have been found between low-density lipoprotein (LDL) peroxidation and the development of atherosclerosis [11,12].

The paraoxonase (*PON*) gene family comprises three isoforms, *PON1*, *PON2* and *PON3*, located in 7q21.3-22.1 [13]. The 60 to 80% structural similarity among these three members accounts for their functional similarity [13,14]. All three isoforms have been implicated as candidate genes for atherosclerosis and cardiovascular diseases due to their ability to attenuate lipid peroxidation, and due to their antioxidant and antiatherogenic effects [15-17]. Low levels of PON activity are thought to increase the risk of atherosclerosis [18], and thereby contribute to a predisposition towards stroke, coronary artery disease (CAD) and vascular disorders in diabetes [19-21]. Other studies have demonstrated a positive association between single nucleotide polymorphisms (SNPs) in *PON* genes and stroke susceptibility [22-25], although conflicting results have been seen in different ethnic groups [26-28]. However, there are limited number of prospective studies validating the association between *PON* genes and the risk of stroke in the Han Chinese population [26,28-30].

A negative association has previously been demonstrated between SNPs in the coding region of *PON1* and *PON2,* and the development of stroke. In this study we wanted to evaluate the levels of ischemic and hemorrhagic risk conferred by SNPs in the whole *PON* family in a large Chinese population. With this aim in mind, we conducted a case–control study in the Han Chinese population to evaluate the possible association of *PON* family genes with total stroke and its subtypes.

## Methods

### Subjects

The study sample included 508 healthy controls and 498 patients, including 328 with ischemic stroke and 170 with hemorrhagic stroke who presented consecutively to the Department of Neurology, Beijing Tiantan Hospital, between December 2010 and March 2011. The subjects were unrelated to one another and were recruited from the Han Chinese population.

Hemorrhagic stroke included hypertensive cerebral hemorrhage and subarachnoid hemorrhage. Patients with hemorrhage due to trauma, tumor, vascular malformation and coagulopathy were excluded. Ischemic stroke was defined as a sudden onset of focal or global neurologic deficit with signs and symptoms persisting for more than 24 h. Patients with a history or occurrence of transient ischemic attack, cerebral embolism, cerebral trauma, cerebrovascular malformations, coagulation disorders, autoimmune diseases, tumors, peripheral vascular disease, or chronic infection diseases were excluded from the study.

All diagnoses were confirmed by brain computed tomography and/or magnetic resonance imaging. The brain images were independently assessed by a technologist and a physician.

Control subjects were recruited from the health examination department of the Beijing Tiantan Hospital. These subjects had no clinical or radiological evidence of stroke and other neurological diseases. They were also free from autoimmune disease, liver disease, nephrosis, and hematological disorders

Sex, age, total plasma cholesterol (TC), triglycerides (TG), HDL, and LDL cholesterol were documented on entry into the study. Potential vascular risk factors were evaluated, including hypertension, diabetes mellitus, atrial fibrillation, and ischemic heart disease. Hypertension was defined according to WHO/ISH criteria [31] as systolic blood pressure ≥140 mmHg and/or diastolic pressure ≥ 90 mmHg with concomitant use of antihypertensive medications Diabetes mellitus was defined as fasting plasma glucose ≥7.0 mmol/L or current treatment with anti-diabetic drugs.

The experimental protocol was approved by the Ethics Committee of the Beijing Tiantan Hospital. Written informed consent was obtained from all participants prior to entering the study.

### Genotyping

Eleven single nucleotide polymorphisms (SNPs) were genotyped. These included: rs662 (Gln192Arg), rs13306698 (Arg160Gly), rs854560 (Leu55Met) in coding region of *PON1*; rs705379 (−107/-108), rs705381 (−160/-162), rs854571 (−824/-832), rs854572 (−907/-909) in the promoter of *PON1*; rs12026 (Ala148Gly) and rs7493 (Ser311Cys) of *PON2*, together with rs2074353 (located in intron) and rs1053275 (Ala99Ala) for *PON3*.

The SNPs were genotyped using the Sequenom Mass ARRAY platform (Sequenom, San Diego, CA) according to the iPLEX Gold Application Guide available at (http://www.sequenom.com/sites/genetic-analysis/applications/snp-genotyping). The genotyping analysis was undertaken according to the manufacturer’s protocol, using recommended reagents in the iPLEX Gold SNP genotyping kit. Briefly, specific assays were designed using the Mass ARRAY Assay Design software package (v3.1). The process involved a locus-specific PCR reaction based on a locus-specific primer extension reaction. Residual nucleotides were dephosphorylated with SAP enzymes before undertaking the iPLEX GOLD primer extension reactions.

Following the single-base extension reactions the products were desalinated with Spectro CLEAN resin (Sequenom). A 10 nL aliquot of the desalinated product was spotted onto a 384-format Spectro CHIP with the Mass ARRAY Nanodispenser. Mass determination was carried out with the MALDI-TOF mass spectrometer and Mass ARRAY Type 4.0 software was used for data acquisition.

SNP genotypes were named using cluster analysis with a default parameter setting. Genotypes were further reviewed manually to correct classification errors caused by clustering artifacts.

#### Statistical analysis

Statistical analysis was undertaken using PLINK software (http://pngu.mgh.harvard.edu/~purcell/plink/) [32]. Hardy-Weinberg equilibrium tests (HWE) were performed for each SNP, and association tests were undertaken using additive, dominant, or recessive genetic models.

Logistic regression was used for risk stratification with or without covariate adjustments determined by significant differences between total stroke patients and controls (i.e. age, HDL, and hypertension). The model with the highest likelihood was considered to provide the best-fit genetic model for each SNP. Haplotype-based association analysis was performed using logistic regression with or without adjustment for covariates. A single site association test between rs662 and rs854560 and ischemic stroke was conducted using an allele-based model. Bonferroni correction was undertaken for the 10 SNPs that were adopted into the single site association analysis.

Linkage disequilibrium analysis and haplotype selection were performed using Haploview software with parameter settings for pairwise tagging with *D’* >0.95 [33]. The Omnibus ANOVA test was conducted using R software [34].

Inverse variance meta-analysis (RevMan 4.0 software) was used to contextualize our studies with two meta-analyses, using the data from PMID: 20856122 [35] and PMID: 18511872 [30], which also studied the association between rs662 and rs854560 loci and ischemic stroke.

Values of *P* <0.005 were considered to represent the threshold for statistical significance.

## Results

### Clinical characteristics of total stroke patients and controls

Table 1 shows demographic characteristics and clinical vascular variables in the control and total stroke patients. There were no significant differences in levels of TC, TG and LDL between the controls and total stroke cases. However, HDL levels were significantly lower in stroke cases than in controls and mean age and incidence of hypertension were significantly higher.

**Table 1:** Comparison of clinical variables between total strokes and control subjects

**Variables**	**Stroke cases (n = 498)**	**Control cases (n = 498)**
Ischemic stroke, n	328	
Hemorrhagic stroke, n	170	
Age, years	60.45 ± 14.27*	56.48 ± 4.55
Male, n (%)	142 (28)	140 (28)
TC, mmol/L	4.41 ± 1.31	4.36 ± 1.33
TG, mmol/L	1.54 ± 0.95	1.56 ± 1.26
HDL, mmol/L	1.10 ± 0.28*	1.28 ± 0.27
LDL, mmol/L	2.54 ± 0.89	2.52 ± 0.56
Hypertension, n (%)	413 (83)*	310 (62)
Diabetes, n (%)	130 (26)	122 (24)

Data are shown as mean ± standard deviation (SD) or as n (%). Abbreviations: TC, total cholesterol; TG, triglycerides; HDL, high-density lipoprotein; LDL, low-density lipoprotein.*Significant differences between cases and controls.

### Linkage disequilibrium

A total of eleven gene polymorphisms were genotyped in the cases and controls. For *PON1* these included three coding-region polymorphisms (rs662/Q192R, rs13306698/Arg160Gly, and rs854560/Leu55Met) and four regulatory-region polymorphisms (rs705379/-107/-108, rs705381/-160/-162, rs854571/-824/-832, and rs854572/-907/-909). There were also two coding-region polymorphisms of *PON2* (rs12026/Ala148Gly, and rs7493/Ser311Cys), and two coding-region polymorphisms of *PON3* (rs2074353 located in intron and rs1053275/Ala99Ala). The total rate of successful genotyping was 98.6%. All genotype distributions within the studied polymorphisms were in Hardy-Weinberg equilibrium (*P* >0.05), in both cases and controls, except for rs705379 (−107/-108) (*P* <0.001), which was located in the promoter of *PON1*.

**Figure 1:**
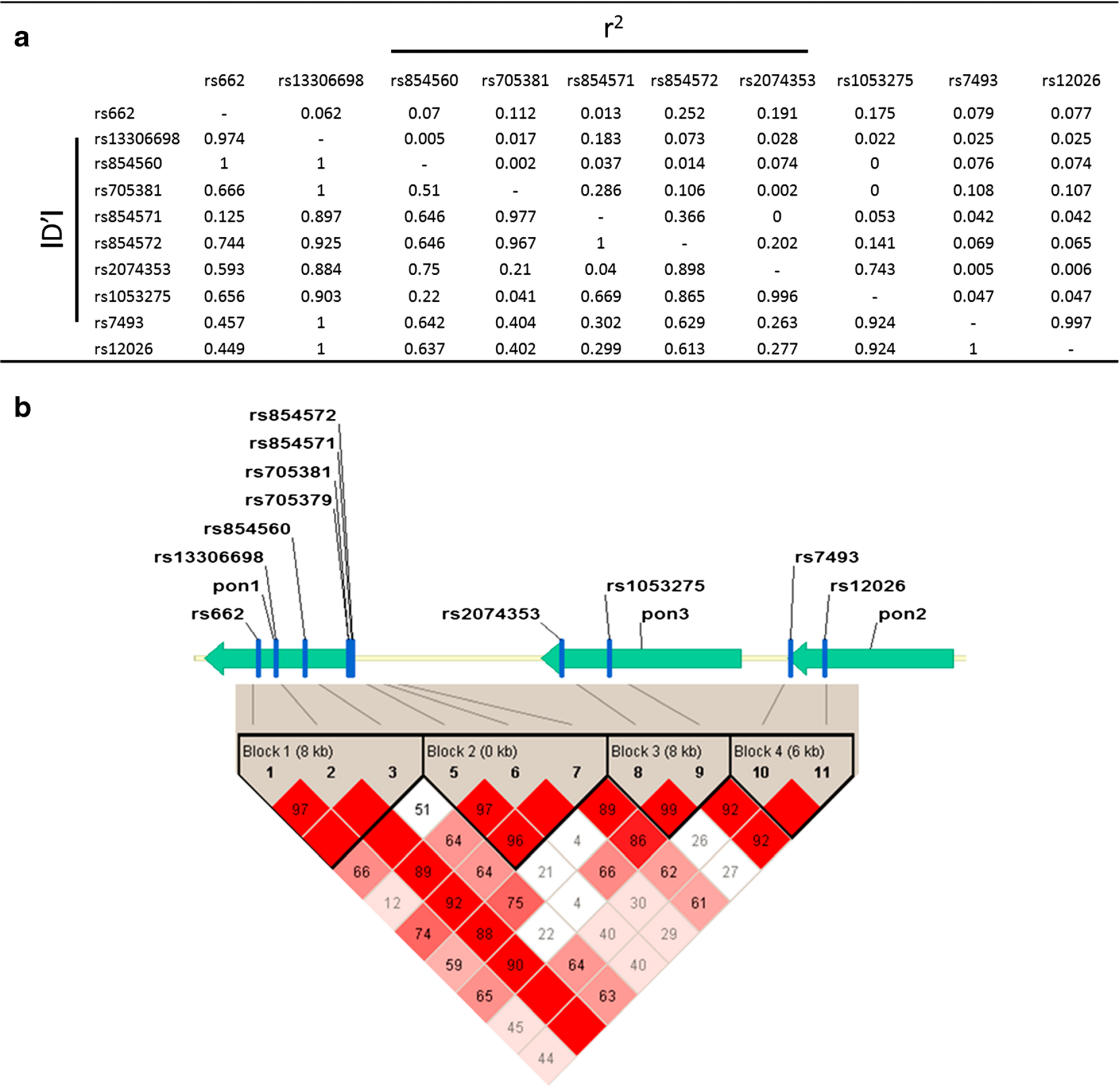
**Linkage disequilibrium analysis of the ten SNPs investigated in healthy controls (a).** Four blocks were identified using Haploview software: Block1 (rs854560-rs13306698-rs662); Block2 (rs854572-rs854571-rs705381); Block3 (rs1053275-rs2074353); Block4 (rs12026-rs7493) (**b**).

The results of linkage disequilibrium evaluation analyses are shown in Figure 1A. In this analysis, SNPs with a pairwise r^2^ >0.9 were considered to be in the same block. Based on this approach, four haplotype blocks (Block1: rs854560-rs13306698-rs662; Block2: rs854572-rs854571-rs705381; Block3: rs1053275-rs2074353; Block4: rs12026-rs7493) were identified (Figure 1B).

### Single site association

The association between the ten SNPs included in the four blocks and total stroke occurrence was analyzed using additive, dominant, genotype, and recessive models. As shown in Table 2, two polymorphisms, rs705381 and rs854571 were significantly associated with total stoke using additive and dominant models. The allele A of rs705381 and the allele T of rs854571 were both less frequent in patients with total stroke than in controls. The association remained significant after logistic regression analysis adjusting for age, HDL and hypertension using the additive model (rs705381, *P*_*adjusted*_ = 0.0058, *OR* = 0.67 [95% CI = 0.50-0.89]; and rs854571, *P*_*adjusted*_ = 0.0330, *OR* = 0.80 [95% CI = 0.65-0.98]). However, both P-values failed to reach significance after the Bonferroni adjustment for multiple comparisons. Analysis using the dominant model, showed that the differences in rs705381 remained significant after Bonferroni correction (*P*_*adjusted*_ = 0.0007, *OR* = 0.57 [95% CI = 0.41-0.79]), but the differences in rs854571 did not. There was no significant association between any of the SNPs of *PON* genes and total strokes when analyzed using the recessive model.

**Table 2:** Association between SNPs and total stroke using the additive, dominant, genotype, and the recessive models

**SNP**	**Model**	**Allele or geno**	**F_Stroke**	**F_Control**	**T-Statistic**		**Logistic Regression**	
					***OR *(95%CI)**	***P***_***unadjusted***_	***OR *(95%CI)**	***P***_***adjusted***_
*rs854571*	*Additive*	C > T	298/992	344/982	0.79(0.65-0.96)	**1.71E-02**	0.80(0.65-0.98)	**3.30E-02**
	*Dominant*	CC + CT/TT	253/496	289/491	0.73(0.57-0.94)	**1.33E-02**	0.75(0.57-0.99)	**3.96E-02**
	*Recessive*	CC/CT + TT	45/496	55/491	0.79(0.52-1.20)	2.69E-01	0.75(0.48-1.17)	2.06E-01
*rs13306698*	*Additive*	A > G	98/1016	98/988	0.97(0.71-1.31)	8.31E-01	1.00(0.71-1.40)	9.99E-01
	*Dominant*	AA + AG/GG	97/508	96/494	0.98(0.71-1.34)	8.92E-01	1.02(0.72-1.44)	9.20E-01
	*Recessive*	AA/AG + GG	1/508	2/494	0.49(0.04-5.37)	5.55E-01	0.39(0.03-5.06)	4.74E-01
*rs854572*	*Additive*	C > G	443/1004	413/964	1.05(0.88-1.26)	5.69E-01	1.09(0.89-1.32)	4.08E-01
	*Dominant*	CC + CG/GG	343/502	324/482	1.05(0.81-1.38)	7.10E-01	1.11(0.82-1.48)	5.04E-01
	*Recessive*	CC/CG + GG	100/502	89/482	1.10(0.80-1.51)	5.62E-01	1.13(0.80-1.61)	4.93E-01
*rs7493*	*Additive*	C > G	192/1016	176/974	1.06(0.84-1.33)	6.32E-01	1.00(0.78-1.29)	9.89E-01
	*Dominant*	CC + CG/GG	173/508	163/487	1.03(0.79-1.34)	8.45E-01	1.00(0.75-1.34)	9.85E-01
	*Recessive*	CC/CG + GG	19/508	13/487	1.42(0.69-2.90)	3.41E-01	1.00(0.46-2.18)	9.93E-01
*rs662*	*Additive*	G > A	389/1014	356/978	1.08(0.91-1.29)	3.86E-01	1.05(0.87-1.28)	5.93E-01
	*Dominant*	GG + GA/AA	303/507	282/489	1.09(0.85-1.40)	5.02E-01	1.05(0.80-1.39)	7.31E-01
	*Recessive*	GG/GA + AA	86/507	74/489	1.15(0.82-1.61)	4.32E-01	1.12(0.77-1.62)	5.65E-01
*rs12026*	*Additive*	C > G	192/1010	174/978	1.09(0.86-1.37)	4.80E-01	1.05(0.81-1.35)	7.17E-01
	*Dominant*	CC + CG/GG	173/505	162/489	1.05(0.81-1.37)	7.07E-01	1.05(0.78-1.40)	7.56E-01
	*Recessive*	CC/CG + GG	19/505	12/489	1.55(0.75-3.24)	2.39E-01	1.13(0.51-2.50)	7.72E-01
*rs1053275*	*Additive*	A > G	203/1000	186/994	1.10(0.89-1.37)	3.82E-01	1.10(0.86-1.39)	4.53E-01
	*Dominant*	AA + AG/GG	179/500	165/497	1.12(0.86-1.46)	3.88E-01	1.13(0.85-1.51)	3.90E-01
	*Recessive*	AA/AG + GG	24/500	21/497	1.14(0.63-2.08)	6.62E-01	1.04(0.54-1.99)	9.17E-01
*rs705381*	*Additive*	G > A	106/988	151/990	0.67(0.51-0.87)	3.13E-03*	0.67(0.50-0.89)	5.80E-03*
	*Dominant*	GG + GA/AA	95/494	144/495	0.58(0.43-0.78)	3.20E-04*	0.57(0.41-0.79)	7.10E-04*
	*Recessive*	GG/GA + AA	11/494	7/495	1.59(0.61-4.13)	3.43E-01	1.64(0.58-4.61)	3.52E-01
*rs2074353*	*Additive*	A > G	253/996	230/982	1.11(0.91-1.36)	3.16E-01	1.12(0.90-1.40)	3.09E-01
	*Dominant*	AA + AG/GG	219/498	197/491	1.17(0.91-1.51)	2.20E-01	1.23(0.93-1.62)	1.46E-01
	*Recessive*	AA/AG + GG	34/498	33/491	1.02(0.62-1.67)	9.47E-01	0.91(0.53-1.57)	7.31E-01
*rs854560*	*Additive*	A > T	41/1014	39/996	1.03(0.66-1.61)	8.84E-01	0.95(0.58-1.56)	8.37E-01
	*Dominant*	AA + AT/TT	40/507	38/498	1.04(0.65-1.65)	8.78E-01	0.97(0.58-1.62)	9.12E-01
	*Recessive*	AA/AT + TT	1/507	1/498	0.98(0.06-15.75)	9.90E-01	0.40(0.02-7.40)	5.39E-01

Variants are described as minor allele or geno; the contrast allele refers to the minor allele; *OR*: odds ratio; CI: confidence interval; *P*_*unjusted*_: unadjusted P-value from t-test; *P*_*adjusted*_: P value adjusted using logistic regression analysis with age, HD and hypertension as covariates. F_Stroke and F_Control represent the frequency of minor allele or geno in total stroke patients and controls respectively. Significant P values (*P* <0.05) are in bold and P* < 0.005 (Bonferroni multiple correction threshold).

As shown in Table 3, rs705381 was significantly associated with ischemic stroke after adjustment of confounders in both additive and dominant models (*P*_*adjusted*_ = 0.0017, *OR* = 0.54 [95% CI = 0.37-0.79]). However, no significant association with ischemic stroke was found using the recessive model.

**Table 3:** Association between SNPs with ischemic stroke using the additive, dominant, genotype, and the recessive models

**SNP**	**Model**	**Allele or geno**	**F_IS**	**Control**	**T-Statistic**		**Logistic Regression**	
					***OR *(95% CI)**	***P***_***unadjusted***_	***OR *(95% CI)**	***P***_***adjusted***_
*rs854571*	*Additive*	C > T	200/660	344/982	0.80(0.65-0.99)	**4.34E-02**	0.84(0.66-1.07)	1.62E-01
	*Dominant*	CC + CT/TT	170/330	289/491	0.74(0.56-0.98)	**3.79E-02**	0.80(0.58-1.10)	1.63E-01
	*Recessive*	CC/CT + TT	30/330	55/491	0.79(0.50-1.27)	3.31E-01	0.82(0.49-1.37)	4.45E-01
*rs13306698*	*Additive*	A > G	62/676	98/988	0.91(0.65-1.29)	6.00E-01	1.07(0.72-1.59)	7.43E-01
	*Dominant*	AA + AG/GG	61/338	96/494	0.91(0.64-1.30)	6.16E-01	1.09(0.72-1.64)	6.83E-01
	*Recessive*	AA/AG + GG	1/338	2/494	0.73(0.07-8.08)	7.98E-01	0.55(0.04-7.85)	6.63E-01
*rs854572*	*Additive*	C > G	285/668	413/964	0.99(0.82-1.21)	9.44E-01	0.98(0.78-1.23)	8.29E-01
	*Dominant*	CC + CG/GG	220/334	324/482	0.94(0.70-1.27)	6.87E-01	0.94(0.67-1.32)	7.23E-01
	*Recessive*	CC/CG + GG	65/334	89/482	1.07(0.75-1.52)	7.21E-01	1.01(0.67-1.53)	9.70E-01
*rs7493*	*Additive*	C > G	124/676	176/974	1.02(0.79-1.32)	8.85E-01	0.98(0.73-1.31)	8.82E-01
	*Dominant*	CC + CG/GG	114/338	163/487	1.01(0.75-1.36)	9.39E-01	0.99(0.71-1.39)	9.68E-01
	*Recessive*	CC/CG + GG	10/338	13/487	1.11(0.48-2.57)	8.04E-01	0.85(0.34-2.12)	7.23E-01
*rs662*	*Additive*	G > A	276/674	356/978	1.19(0.98-1.45)	7.34E-02	1.18(0.94-1.47)	1.46E-01
	*Dominant*	GG + GA/AA	212/337	282/489	1.25(0.94-1.66)	1.31E-01	1.20(0.86-1.66)	2.84E-01
	*Recessive*	GG/GA + AA	64/337	74/489	1.32(0.91-1.90)	1.45E-01	1.35(0.88-2.06)	1.67E-01
*rs12026*	*Additive*	C > G	124/672	174/978	1.05(0.81-1.36)	7.26E-01	1.02(0.76-1.37)	8.78E-01
	*Dominant*	CC + CG/GG	114/336	162/389	1.04(0.77-1.39)	8.11E-01	1.04(0.74-1.45)	8.33E-01
	*Recessive*	CC/CG + GG	10/336	12/489	1.22(0.52-2.86)	6.48E-01	0.95(0.38-2.42)	9.21E-01
*rs1053275*	*Additive*	A > G	144/666	186/994	1.19(0.94-1.52)	1.51E-01	1.17(0.89-1.54)	2.70E-01
	*Dominant*	AA + AG/GG	129/333	165/497	1.27(0.95-1.70)	1.02E-01	1.24(0.89-1.73)	2.10E-01
	*Recessive*	AA/AG + GG	15/333	21/497	1.07(0.54-2.11)	8.47E-01	1.07(0.49-2.31)	8.68E-01
*rs705381*	*Additive*	G > A	74/660	151/990	0.70(0.52-0.95)	**2.02E-02**	0.65(0.47-0.92)	**1.35E-02**
	*Dominant*	GG + GA/AA	65/330	144/395	0.60(0.43-0.83)	2.50E-03*	0.54(0.37-0.79)	1.67E-03*
	*Recessive*	GG/GA + AA	9/330	7/495	1.96(0.72-5.30)	1.88E-01	1.85(0.60-5.64)	2.83E-01
*rs2074353*	*Additive*	A > G	176/662	230/982	1.18(0.94-1.47)	1.53E-01	1.23(0.95-1.60)	1.09E-01
	*Dominant*	AA + AG/GG	153/331	197/491	1.28(0.97-1.70)	8.30E-02	1.38(1.00-1.91)	5.29E-02
	*Recessive*	AA/AG + GG	23/331	33/491	1.04(0.60-1.80)	8.99E-01	1.06(0.56-1.99)	8.69E-01
*rs854560*	*Additive*	A > T	30/674	39/996	1.14(0.70-1.85)	5.93E-01	1.19(0.69-2.07)	5.36E-01
	*Dominant*	AA + AT/TT	29/337	38/498	1.14(0.69-1.89)	6.11E-01	1.24(0.70-2.21)	4.57E-01
	*Recessive*	AA/AT + TT	1/337	1/498	1.48(0.09-23.73)	7.82E-01	0.43(0.02-9.62)	5.92E-01

Variants are described as minor allele or geno and the contrast allele refers to the minor allele; *OR*: odds ratio; CI: confidence interval; *P*_*unjusted*_: unadjusted P-value from t-test; *P*_*adjusted*_: P value adjusted using logistic regression analysis with age, HD and hypertension as covariates F_IS and F_Control represent the frequency of minor allele in ischemic stroke patients and controls respectively. Significant P values (*P* < 0.05) are in bold and P* < 0.*005* (Bonferroni multiple correction threshold).

Rs854571 was associated with hemorrhagic stroke, with marginal significance (*P*_*unadjusted*_ = 0.0500, *OR* = 0.76 [95% CI = 0.57-1.00]) using the additive model, and rs705381 showed a significant association in both additive (*P*_*adjusted*_ = 0.0290, *OR* = 0.62 [95% CI = 0.40-0.95]) and dominant models (*P*_*adjusted*_ = 0.0165, *OR* = 0.57 [95% CI = 0.36-0.90]) (Table 4). However, neither of the two SNPs was significantly associated with hemorrhagic stroke after the Bonferroni correction. Thus, there was no significant finding for hemorrhagic stroke with any of the three models.

**Table 4:** Association between SNPs and hemorrhagic stroke using the additive, dominant, genotype, and the recessive models

**SNP**	**Model**	**Allele or geno**	**F_HS**	**F_Control**	**T-Statistic**		**Logistic Regression**	
					***OR *(95% CI)**	***P***_***unadjusted***_	***OR *(95% CI)**	***P***_***adjusted***_
*rs854571*	*Additive*	C > T	92/316	344/982	0.76(0.57-1.00)	5.00E-02	0.76(0.57-1.01)	5.54E-02
	*Dominant*	CC + CT/TT	78/158	289/491	0.68(0.48-0.98)	3.68E-02	0.70(0.48-1.01)	5.57E-02
	*Recessive*	CC/CT + TT	14/158	55/491	0.77(0.42-1.43)	4.08E-01	0.71(0.38-1.34)	2.95E-01
*rs13306698*	*Additive*	A > G	33/324	98/988	1.03(0.67-1.59)	8.85E-01	1.06(0.68-1.66)	7.93E-01
	*Dominant*	AA + AG/GG	33/162	96/494	1.06(0.68-1.65)	7.95E-01	1.09(0.69-1.72)	7.06E-01
	*Recessive*	AA/AG + GG	0/162	2/494	0.00(0.00-inf)	9.99E-01	0.00(0.00-inf)	9.99E-01
*rs854572*	*Additive*	C > G	151/320	413/964	1.20(0.93-1.54)	1.73E-01	1.24(0.95-1.61)	1.20E-01
	*Dominant*	CC + CG/GG	118/160	324/482	1.37(0.92-2.04)	1.23E-01	1.38(0.91-2.08)	1.27E-01
	*Recessive*	CC/CG + GG	33/160	89/482	1.15(0.73-1.79)	5.46E-01	1.25(0.79-1.99)	3.38E-01
*rs7493*	*Additive*	C > G	64/324	176/974	1.12(0.81-1.55)	4.94E-01	1.05(0.75-1.46)	7.77E-01
	*Dominant*	CC + CG/GG	57/162	163/487	1.08(0.74-1.57)	6.90E-01	1.03(0.70-1.51)	8.94E-01
	*Recessive*	CC/CG + GG	7/162	13/487	1.65(0.65-4.20)	2.97E-01	1.28(0.49-3.36)	6.13E-01
*rs662*	*Additive*	G > A	108/324	356/978	0.88(0.68-1.14)	3.36E-01	0.85(0.65-1.12)	2.48E-01
	*Dominant*	GG + GA/AA	88/162	282/489	0.87(0.61-1.25)	4.56E-01	0.82(0.56-1.18)	2.85E-01
	*Recessive*	GG/GA + AA	20/162	74/489	0.79(0.47-1.34)	3.83E-01	0.80(0.46-1.38)	4.26E-01
*rs12026*	*Additive*	C > G	64/322	174/978	1.15(0.83-1.59)	3.95E-01	1.10(0.78-1.53)	5.92E-01
	*Dominant*	CC + CG/GG	57/161	162/389	1.11(0.76-1.61)	5.96E-01	1.07(0.72-1.57)	7.44E-01
	*Recessive*	CC/CG + GG	7/161	12/489	1.81(0.70-4.67)	2.22E-01	1.47(0.55-3.92)	4.39E-01
*rs1053275*	*Additive*	A > G	58/318	186/994	0.97(0.71-1.33)	8.55E-01	0.96(0.70-1.33)	8.07E-01
	*Dominant*	AA + AG/GG	49/159	165/497	0.90(0.61-1.32)	5.77E-01	0.91(0.61-1.35)	6.36E-01
	*Recessive*	AA/AG + GG	9/159	21/497	1.36(0.61-3.03)	4.53E-01	1.17(0.51-2.69)	7.09E-01
*rs705381*	*Additive*	G > A	32/312	151/990	0.62(0.41-0.94)	2.42E-02	0.62(0.40-0.95)	2.90E-02
	*Dominant*	GG + GA/AA	30/156	144/395	0.58(0.37-0.90)	1.61E-02	0.57(0.36-0.90)	1.65E-02
	*Recessive*	GG/GA + AA	2/156	7/495	0.91(0.19-4.40)	9.02E-01	1.22(0.24-6.22)	8.13E-01
*rs2074353*	*Additive*	A > G	74/340	230/982	0.99(0.74-1.32)	9.57E-01	0.96(0.72-1.29)	8.05E-01
	*Dominant*	AA + AG/GG	63/159	197/491	0.98(0.68-1.41)	9.11E-01	0.98(0.67-1.42)	8.98E-01
	*Recessive*	AA/AG + GG	11/159	33/491	1.03(0.51-2.09)	9.31E-01	0.87(0.42-1.82)	7.21E-01
*rs854560*	*Additive*	A > T	9/324	39/996	0.70(0.34-1.46)	3.46E-01	0.57(0.26-1.25)	1.58E-01
	*Dominant*	AA + AT/TT	9/162	38/498	0.71(0.34-1.51)	3.74E-01	0.57(0.26-1.28)	1.74E-01
	*Recessive*	AA/AT + TT	0/162	NA	NA	NA	NA	NA

Variants are described as minor allele or geno and the contrast allele refers to the minor allele; *P*_*unjusted*_: unadjusted P-value from t-test; *P*_*adjusted*_: P value adjusted using logistic regression analysis with age, HD and hypertension as covariates. F_HS and F_Control represent the frequency of minor allele in hemorrhagic stroke patients and controls respectively. NA means not applicable. Significant P values (*P* < 0.05) are in bold.

### Haplotype analysis

Haplotype analysis conducted in the four blocks, with or without adjustment for age, HDL and hypertension as covariates is shown in Table 5. Block 2 consisting of rs854572, rs854571 and rs705381 was associated with total stroke (*P* = 0.0129 Omnibus test), and included one protective haplotype C-T-C (*OR* = 0.64; *P*_*unadjusted*_ = 0.0013,) and one nominal risk haplotype C-C-C (*OR* = 1.24; *P*_*unadjusted*_ = 0.0442,). The association for haplotype C-T-C remained significant after adjustment for age, HDL and hypertension as covariates (*OR* = 0.65; *P* = 0.0037). No other significant haplotype associations were found.

**Table 5:** Haplotypes of the four blocks between total strokes and control subjects

**Haplotype**	**Logistic Regression**			
	***OR***	***P***_***unadjusted***_	***OR***	***P***_***adjusted***_
**Block1: rs854560-rs13306698-rs662**				
OMNIBUS	NA	0.9371	NA	0.9569
TAA	1.03	0.8820	0.95	0.8390
AAA	1.08	0.4170	1.06	0.5790
AGG	0.95	0.7640	0.98	0.8840
AAG	0.94	0.4810	0.96	0.6580
**Block2: rs854572-rs854571-rs705381**				
OMNIBUS	NA	**0.0129**	NA	**0.0394**
CTT	1.05	0.6170	1.08	0.4200
CTC	0.64	**0.0013**	0.65	**0.0037**
GCC	0.99	0.9110	0.99	0.9280
CCC	1.24	**0.0442**	1.19	0.1420
**Block3: rs1053275-rs2074353**				
OMNIBUS	NA	0.4970	NA	0.5757
GG	1.10	0.3970	1.10	0.4210
AG	1.09	0.6630	1.13	0.5880
AA	0.90	0.2920	0.89	0.3010
**Block4: rs12026-rs7493**				
OMNIBUS	NA	0.2479	NA	0.5467
GG	1.08	0.5390	1.03	0.8430
CC	0.92	0.5020	0.96	0.7660

Haplotypes observed in <1% of the control subjects are not listed in the table. *OR*: odds ratio; *P*_*unjusted*_: unadjusted P-value from t-test; *P*_*adjusted*_: P value adjusted using logistic regression analysis with age, HD and hypertension as covariates. OMIBUS value was calculated by an ANOVA analysis for including or not including the haplotype information in a likelihood ration test of nested model. The OR in one block for each haplotype was calculated by using all the other haplotypes in the same block as the reference haplotype. Significant P values (*P* < 0.05) are in bold.

### Meta-analysis

**Figure 2:**
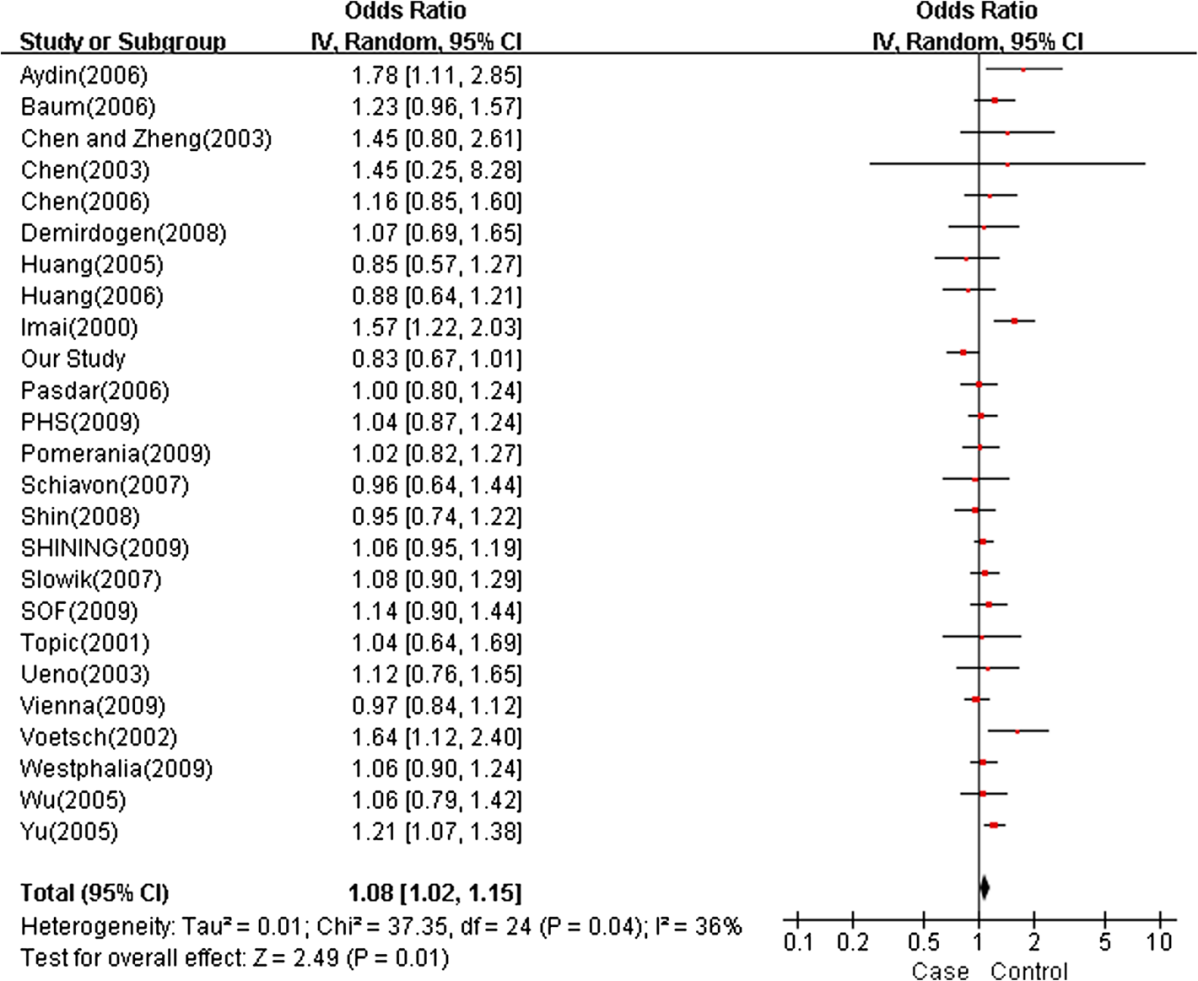
**Meta-analysis of studies investigating the association of PON1 rs662 with ischemic stroke using a random effects model.** The point estimate of the *OR* (square proportional to the weight of each study) and 95% *CI* for the *OR* (extending lines) for each study. The summary *OR* and 95% *CIs* by random effects calculations are depicted as a diamond. Values higher than 1 indicate that the G allele is associated with increased risk of ischemic stroke.

Two meta-analyses, PMID: 20856122 [35] and PMID: 18511872 [30], which studied the association between rs662 and rs854560 loci and ischemic stroke were contextualized with our study using the random effects model. Forests plot for rs662 from 25 studies including our own are shown in Figure 2. There was a nominal significant association between rs662 and ischemic stroke (*P* = 0.0100, *OR* = 1.08 [95% CI = 1.02-1.15]) yielding 1.08 per G allele copy, with no statistical evidence for statistical heterogeneity (*P* = 0.0400, *I*^*2*^ = 36%) between studies.

**Figure 3:**
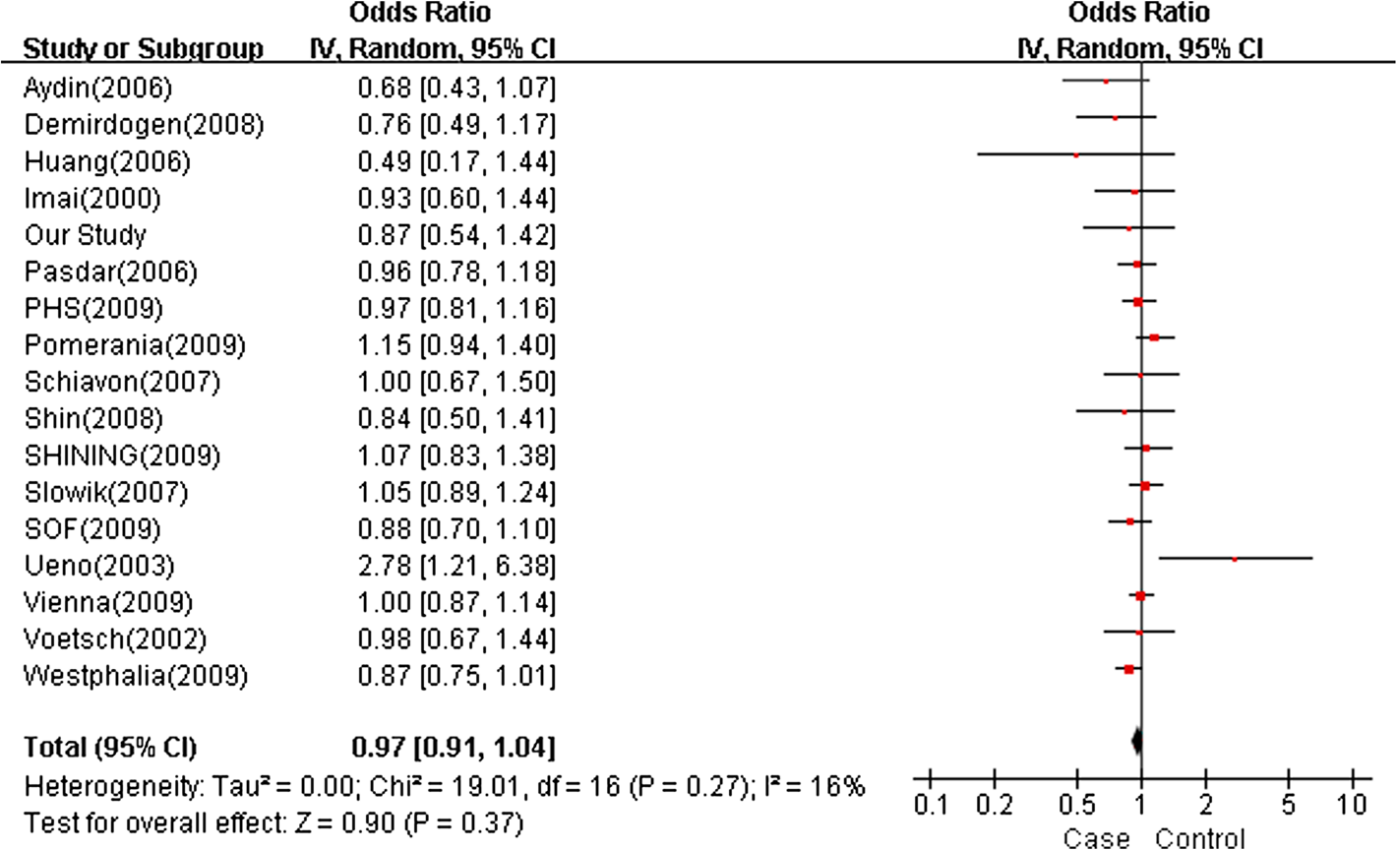
**Meta-analysis of studies investigating the association of PON1 rs854560 with ischemic stroke using a random effects model.** Values higher than 1 indicate that the A allele is associated with increased risk of ischemic stroke risk. The layout is the same as that in Figure 2.

There was no evidence of an association between rs854560 and ischemic stroke risk (*P* = 0.3700, *OR* = 0.97 [95% CI = 0.91-1.04]) and no evidence of heterogeneity (*P* = 0.2700, *I*^*2*^ = 16%) between studies (Figure 3).

## Discussion

The present study investigated the association of 11 polymorphisms in 3 *PON* genes with the risk of stroke. Using a dominant model, we demonstrated that rs705381 (−162) was significantly associated with total stroke and ischemic stroke but not with hemorrhagic stroke. There was also a nominal association between rs854571 (−824) and stroke with the allele T as a protective factor.

Both rs705381 and rs854571 polymorphisms located in the promoter region of *PON1* were associated with stroke, which was consistent with previous findings [19,36-39]. The protective effect of -162 T polymorphism on total stroke and ischemic stroke was also consistent with previous observations [40] which suggested that NF-1, a ubiquitous nuclear factor and a transcriptional activator, has a binding site on *PON1* if allele A appears at −162. Other studies have shown that -162 T polymorphism results in higher expression levels of *PON1*[40,41] There is also evidence to suggest a correlation between AA (−162) and high PON activities in Caucasians [42].

Our results support the hypothesis that the protective effect of -162 T polymorphism might be attributable to high PON activity [42]. We also found weak evidence to suggest that -824 T was associated with a reduced propensity to suffer stroke. However, the evidence was no longer apparent after Bonferroni correction for multiple comparisons. It has been previously reported that -824 T (824A in their finding) was associated with low serum PON levels [43]. Negative associations between −162 and −824 have been reported in studies in American populations [23,40]. These findings highlight the potential influence of ethnic differences in terms of the founder effect and identical-by-descent principles [44,45].

Patients with coronary heart disease (CHD) have been shown to have a higher frequency of -162 T allele than the controls, suggesting allele A may be associated with risk of CHD in the Han Chinese population [46]. However, in our study, we found a protective effect of the -162 T polymorphism on stroke.

Haplotype analysis further confirmed our positive results and identified a positive association between the protective haplotype C-T-C and the risk haplotype C-C-C of rs854572-rs854571-rs705381 (Block 2) with total stroke. No significant associations were observed for stroke susceptibility with the two coding region polymorphisms in *PON2*, which was consistent with previous findings in the Han Chinese population and in North Americans [24,29], although a positive association of Ser311Cys was found in a Polish population [22].

The absence of any positive correlations between stroke risk and the two *PON3* polymorphisms in our study was also consistent with reported findings in Caucasian and North American patients [24,27].

Our study was conducted in a relatively large Chinese sample pool and included careful assessment of two stroke subtypes. We also selected common variants in all three members of the PON gene family. However, functional detection of PON activities was not undertaken in the present study and investigation of the association between SNPs and large or small vessel strokes was not possible as a complete classification of the subtype of ischemic stroke subjects was not available in our study. In our study, results from both adjusted and unadjusted analyses were in line with each other. However, in other settings, authorities have discouraged the use of data adjustments for the determination of the total genetic effect [47]. It, therefore, remains uncertain as to whether adjusted or unadjusted data should be used to interpret our results in clinical context.

## Conclusion

The study identified rs705381 (−162) as being significantly associated with total stroke and ischemic stroke, and demonstrated a weak association for rs854571 (−824) in the Han Chinese population. These findings support the involvement of *PON* polymorphisms in the development of stroke. Further studies with larger sample sizes are required to validate these findings and to elucidate the underlying biological mechanisms.

## Competing interests

The authors have no competing interests.

## Authors’ contributions

YS and YS designed the study, coordinated sample recruitment and revised the final manuscript. GZ participated in study design and collected the samples. WL drafted the manuscript and carried out the statistical analysis. ZL helped with the statistical analysis and draft manuscript preparation. HL, RM and XK helped with the sample collection. YR and XL performed the SNP genotyping. All authors read and approved the final manuscript.

## Pre-publication history

The pre-publication history for this paper can be accessed here:

http://www.biomedcentral.com/1471-2350/14/16/prepub

## References

[B1] Adams H Bendixen B Kappelle L Biller J Love B Gordon D Marsh E Classification of subtype of acute ischemic stroke. Definitions for use in a multicenter clinical trial. TOAST. Trial of Org 10172 in Acute Stroke Treatment Stroke 1993 24 1 35 41 7678184 10.1161/01.str.24.1.35

[B2] Liao DP Myers R Hunt S Shahar E Paton C Burke G Province M Heiss G Familial history of stroke and stroke risk - The family heart study Stroke 1997 28 10 1908 1912 9341694 10.1161/01.str.28.10.1908

[B3] de Lange M Snieder H Ariens RAS Spector TD Grant PJ The genetics of haemostasis: a twin study Lancet 2001 357 9250 101 105 11197396 10.1016/S0140-6736(00)03541-8

[B4] Bak S Gaist D Sindrup SH Skytthe A Christensen K Genetic liability in stroke - a long-term follow-up study of Danish twins Stroke 2002 33 3 769 774 11872902 10.1161/hs0302.103619

[B5] Rubattu S Volpe M Kreutz R Ganten U Ganten D Lindpaintner K Chromosomal mapping of quantitative trait loci contributing to stroke in a rat model of complex human disease Nat Genet 1996 13 4 429 434 8696337 10.1038/ng0896-429

[B6] Jeffs B Clark JS Anderson NH Gratton J Brosnan MJ Gauguier D Reid JL Macrae IM Dominiczak AF Sensitivity to cerebral ischaemic insult in a rat model of stroke is determined by a single genetic locus Nat Genet 1997 16 4 364 367 9241273 10.1038/ng0897-364

[B7] Joutel A Corpechot C Ducros A Vahedi K Chabriat H Mouton P Alamowitch S Domenga V Cecillion M Marechal E Notch3 mutations in CADASIL, a hereditary adult-onset condition causing stroke and dementia Nature 1996 383 6602 707 710 8878478 10.1038/383707a0

[B8] Petersen NH Schmied AB Zeller JA Plendl H Deuschl G Zunker P Lp(a) lipoprotein and plasminogen activity in patients with different etiology of ischemic stroke Cerebrovasc Dis 2007 23 2–3 188 193 17143002 10.1159/000097640

[B9] Lernfelt B Forsberg M Blomstrand C Mellstrom D Volkmann R Cerebral atherosclerosis as predictor of stroke and mortality in representative elderly population Stroke 2002 33 1 224 229 11779914 10.1161/hs0102.102009

[B10] Nagai Y Kitagawa K Sakaguchi M Shimizu Y Hashimoto H Yamagami H Narita M Ohtsuki T Hori M Matsumoto M Significance of earlier carotid atherosclerosis for stroke subtypes Stroke 2001 32 8 1780 1785 11486105 10.1161/01.str.32.8.1780

[B11] Gordon DJ Rifkind BM High-density lipoprotein — the clinical implications of recent studies N Eng J Med 1989 321 19 1311 1316 10.1056/NEJM198911093211907 2677733

[B12] Kozarsky KF Donahee MH Glick JM Krieger M Rader DJ Gene transfer and hepatic overexpression of the HDL receptor SR-BI reduces atherosclerosis in the cholesterol-fed LDL receptor-deficient mouse Arterioscler Thromb Vasc Biol 2000 20 3 721 727 10712397 10.1161/01.atv.20.3.721

[B13] Primo-Parmo SL Sorenson RC Teiber J Du BNL The human serum paraoxonase/arylesterase gene (PON1) is one member of a multigene family Genomics 1996 33 3 498 507 8661009 10.1006/geno.1996.0225

[B14] Reddy ST Wadleigh DJ Grijalva V Ng C Hama S Gangopadhyay A Shih DM Lusis AJ Navab M Fogelman AM Human paraoxonase-3 is an HDL-associated enzyme with biological activity similar to paraoxonase-1 protein but is not regulated by oxidized lipids Arterioscler Thromb Vasc Biol 2001 21 4 542 547 11304470 10.1161/01.atv.21.4.542

[B15] Mackness MI Arrol S Durrington PN Paraoxonase prevents accumulation of lipoperoxides in low-density lipoprotein FEBS Lett 1991 286 1–2 152 154 1650712 10.1016/0014-5793(91)80962-3

[B16] Aviram M Rosenblat M Bisgaier CL Newton RS Primo-Parmo SL La Du BN Paraoxonase inhibits high-density lipoprotein oxidation and preserves its functions - a possible peroxidative role for paraoxonase J Clin Invest 1998 101 8 1581 1590 9541487 10.1172/JCI1649 PMC508738

[B17] Tward A Xia Y-R Wang X-P Shi Y-S Park C Castellani LW Lusis AJ Shih DM Decreased atherosclerotic lesion formation in human serum paraoxonase transgenic mice Circulation 2002 106 4 484 490 12135950 10.1161/01.cir.0000023623.87083.4f

[B18] Mackness MI Arrol S Abbott CA Durrington PN Is paraoxonase related to atherosclerosis Chem Biol Interact 1993 87 1–3 161 171 8393738 10.1016/0009-2797(93)90038-z

[B19] Kim NS Kang K Cha MH Kang BJ Moon J Kang BK Yu BC Kim YS Choi SM Bang OS Decreased paraoxonase-1 activity is a risk factor for ischemic stroke in Koreans Biochem Biophys Res Commun 2007 364 1 157 162 17936248 10.1016/j.bbrc.2007.09.119

[B20] Michalak S Kazmierski R Hellmann A Wysocka E Kocialkowska-Adamczewska D Wencel-Warot A Nowinski WL Serum paraoxonase/arylesterase activity affects outcome in ischemic stroke patients Cerebrovasc Dis 2011 32 2 124 132 21778709 10.1159/000328227

[B21] Martinelli N Micaglio R Consoli L Guarini P Grison E Pizzolo F Friso S Trabetti E Pignatti PF Corrocher R Low levels of serum paraoxonase activities are characteristic of metabolic syndrome and may influence the metabolic-syndrome-related risk of coronary artery disease Exp Diabetes Res 2012 2012 231502 21960992 10.1155/2012/231502 PMC3179885

[B22] Slowik A Wloch D Szermer P Wolkow P Malecki M Pera J Turaj W Dziedzic T Klimkowicz-Mrowiec A Kopec G Paraoxonase 2 gene C311S polymorphism is associated with a risk of large vessel disease stroke in a Polish population Cerebrovasc Dis 2007 23 5–6 395 400 17406108 10.1159/000101462

[B23] Voetsch B Benke KS Panhuysen CI Damasceno BP Loscalzo J The combined effect of paraoxonase promoter and coding region polymorphisms on the risk of arterial ischemic stroke among young adults Arch Neurol 2004 61 3 351 356 15023811 10.1001/archneur.61.3.351

[B24] Ranade K Kirchgessner TG Iakoubova OA Devlin JJ DelMonte T Vishnupad P Hui L Tsuchihashi Z Sacks FM Sabatine MS Evaluation of the paraoxonases as candidate genes for stroke - Gln192Arg polymorphism in the paraoxonase 1 gene is associated with increased risk of stroke Stroke 2005 36 11 2346 2350 16239632 10.1161/01.STR.0000185703.88944.7d

[B25] Voetsch B Benke KS Damasceno BP Siqueira LH Loscalzo J Paraoxonase 192 Gln - > Arg polymorphism - an independent risk factor for nonfatal arterial ischemic stroke among young adults Stroke 2002 33 6 1459 1464 12052975 10.1161/01.str.0000016928.60995.bd

[B26] Xiao ZJ Chen J Sun Y Zheng ZJ Lack of association between the Paraoxonase 1 Q/R192 single nucleotide polymorphism and stroke in a Chinese cohort Acta Neurol Belg 2009 109 3 205 209 19902814

[B27] Pasdar A Ross-Adams H Cumming A Cheung J Whalley L St Clair D MacLeod MJ Paraoxonase gene polymorphisms and haplotype analysis in a stroke population BMC Med Genet 2006 7 28 16551349 10.1186/1471-2350-7-28 PMC1435875

[B28] Huang Q Liu Y-h Yang Q-d Xiao B Ge L Zhang N Xia J Zhang L Liu Z-j Human serum paraoxonase gene polymorphisms, Q192R and L55M, are not associated with the risk of cerebral infarction in Chinese Han population Neurolog Res 2006 28 5 549 554 10.1179/016164106X110337 16808888

[B29] Xu HW Yuan N Zhao Z Zhang L Xia J Zeng KM Xiao B Yang XS Tang BS Study of the relationship between gene polymorphisms of paraoxonase 2 and stroke in a chinese population Cerebrovasc Dis 2008 25 1–2 87 94 18063859 10.1159/000111996

[B30] Xu XW Li JJ Sheng WL Liu L Meta-analysis of genetic studies from journals published in China of ischemic stroke in the Han Chinese population Cerebrovasc Dis 2008 26 1 48 62 18511872 10.1159/000135653

[B31] Afridi I Canny J Yao CH Christensen B Cooper RS Kadiri S Hill S Kaplan N Kuschnir E Lexchin J World Health Organization (WHO)/International Society of Hypertension (ISH) statement on management of hypertension J Hypertens 2003 21 11 1983 1992 14597836 10.1097/00004872-200311000-00002

[B32] Purcell S Neale B Todd-Brown K Thomas L Ferreira MAR Bender D Maller J Sklar P de Bakker PIW Daly MJ PLINK: a tool set for whole-genome association and population-based linkage analyses The Am J Human Genetics 2007 81 3 559 575 10.1086/519795 PMC1950838 17701901

[B33] Barrett JC Fry B Maller J Daly MJ Haploview: analysis and visualization of LD and haplotype maps Bioinformatics 2005 21 2 263 265 15297300 10.1093/bioinformatics/bth457

[B34] Burkett K Graham J McNeney B Hapassoc: software for likelihood inference of trait associations with SNP haplotypes and other attributes J Stat Softw 2006 16 2 1 19

[B35] Dahabreh IJ Kitsios GD Kent DM Trikalinos TA Paraoxonase 1 polymorphisms and ischemic stroke risk: a systematic review and meta-analysis Genet Med 2010 12 10 606 615 20856122 10.1097/GIM.0b013e3181ee81c6 PMC3081717

[B36] Leviev N Righetti A James RW Paraoxonase promoter polymorphism T(−107)C and relative paraoxonase deficiency as determinants of risk of coronary artery disease J Mol Med 2001 79 8 457 463 11511976 10.1007/s001090100240

[B37] Deakin S Leviev I Brulhart-Meynet MC James RW Paraoxonase-1 promoter haplotypes and serum paraoxonase: a predominant role for polymorphic position-107, implicating the Sp1 transcription factor Biochem Jl 2003 372 643 649 12639220 10.1042/BJ20021670 PMC1223427

[B38] Demirdogen BC Demirkaya S Turkanoglu A Bek S Arinc E Adali O Analysis of paraoxonase 1 (PON1) genetic polymorphisms and activities as risk factors for ischemic stroke in Turkish population Cell Biochem Funct 2009 27 8 558 567 19902425 10.1002/cbf.1607

[B39] Karakaya A Ibis S Kural T Kose SK Karakaya AE Serum paraoxonase activity and phenotype distribution in Turkish subjects with coronary heart disease and its relationship to serum lipids and lipoproteins Chemico-Biol Interact 1999 118 3 193 200 10.1016/s0009-2797(99)00085-x 10362226

[B40] Brophy VH Hastings MD Clendenning JB Richter RJ Jarvik GP Furlong CE Polymorphisms in the human paraoxonase (PON1) promoter Pharmacogenetics 2001 11 1 77 84 11207034 10.1097/00008571-200102000-00009

[B41] Brophy VH Jampsa RL Clendenning JB McKinstry LA Jarvik GP Furlong CE Effects of 5 ' regulatory-region polymorphisms on paraoxonase-gene (PON1) expression Am J Hum Genetics 2001 68 6 1428 1436 11335891 10.1086/320600 PMC1226129

[B42] Hofer SE Bennetts B Chan AK Holloway B Karschimkus C Jenkins AJ Silink M Donaghue KC Association between PON 1 polymorphisms, PON activity and diabetes complications Jf Diabetes Complicats 2006 20 5 322 328 10.1016/j.jdiacomp.2005.08.008 16949520

[B43] Leviev I James RW Promoter polymorphisms of human paraoxonase PON1 gene and serum paraoxonase activities and concentrations Arterioscler Thromb Vasc Biol 2000 20 2 516 521 10669651 10.1161/01.atv.20.2.516

[B44] Freedman ML Reich D Penney KL McDonald GJ Mignault AA Patterson N Gabriel SB Topol EJ Assessing the impact of population stratification on genetic association studies Nat Genet 2004 36 4 388 493 15052270 10.1038/ng1333

[B45] Du R Genetics of Chinese population Beijing Sci Publish 2004 10 414 419

[B46] Wang X Fan Z Huang J Su S Yu Q Zhao J Hui R Yao Z Shen Y Qiang B Extensive association analysis between oolymorphisms of PON gene cluster with coronary heart disease in Chinese Han population Arterioscle Tthromb Vasc Biol 2003 23 2 328 334 10.1161/01.atv.0000051702.38086.c1 12588779

[B47] Lash TL Lien EA Sørensen HT Hamilton-Dutoit S Genotype-guided tamoxifen therapy: time to pause for reflection? Lancet Oncol 2009 10 8 825 833 19647203 10.1016/S1470-2045(09)70030-0 PMC2895727

